# Superoperator Master Equations and Effective Dynamics

**DOI:** 10.3390/e26010014

**Published:** 2023-12-22

**Authors:** Alexander Evgen’evich Teretenkov

**Affiliations:** Department of Mathematical Methods for Quantum Technologies, Steklov Mathematical Institute of Russian Academy of Sciences, ul. Gubkina 8, Moscow 119991, Russia; taemsu@mail.ru

**Keywords:** projection method, quantum master equation, effective generator

## Abstract

We developed the projection method to derive an analog of the quantum master equation for propagators rather than density matrices themselves. As these propagators are superoperators, we call them superoperator master equations. Furthermore, as the projector maps superoperators to superoperators, we call it a hyperprojector. We gave general perturbative expansions for generators of the weak coupling superoperator master equation and the stroboscopic limit superoperator master equation. After that, we considered a particular example of a hyperprojector that is the infinite time average of unitary dynamics. We call it the averaging hyperprojector. We discussed the properties of this hyperprojector and its physical meaning. Then, we illustrated our general second order superoperator master equations arising in the weak coupling limit and the stroboscopic limit, taking the averaging hyperprojector as an example, which we call effective dynamics. We discussed some properties of these superoperator master equations, in particular, the entropy increase.

## 1. Introduction

The Nakajima–Zwanzig projection approach [1,2] is wide-spread for the derivation of master equations in statistical physics. It can be used both to derive Nakajima–Zwanzig integro-differential master equations or time-convolutionless master equations [3]. In this work, we are mostly interested in time-convolutionless master equations, as they are widely used in open quantum systems theory [4,5,6,7]. Usually, the projectors are applied to density matrix dynamics to obtain the master equation for such projected density matrices. The difference in our approach is that we apply projectors to dynamical maps and derive master equations for the projected dynamical maps. Therefore, we refer to these equations as superoperator master equations (because these dynamical maps are superoperators) and to the projectors as hyperprojectors (because they map superoperators to superoperators). We apply this technique to the hyperprojector of averaging with respect to free dynamics to obtain an effective dynamical map. Thus, such a map can be considered a dynamical analog of the effective Gibbs state introduced in [8].

We have already used a special case of the hyperprojector technique before to derive effective Heisenberg equations for a specific model with a quadratic fermionic Hamiltonian [9]. However, we have neither discussed a general approach nor given a systematic perturbative expansion for the time-convolutionless weak coupling master equations. Therefore, in Section 2, we describe a general setup of the hyperprojector technique for general dynamical maps for some linear equations. For this purpose, we first briefly summarize the usual projection approach for deriving the time-convolutionless master equations. Then, we introduce our approach in the same notation to emphasize the similarities and differences. In particular, by choosing identity preserving hyperprojectors, the superoperator master equation becomes homogeneous for arbitrary dynamical maps. In the case of the usual projectors, it is not possible for arbitrary density matrices, which leads to non-homogeneous terms in the usual master equations [10], and there are some approaches [11] that are trying to avoid presence of these terms to obtain better convergence. After that, we gave a systematic perturbative expansion for the generators of time-convolutionless weak coupling master equations. In Section 2.1, we focus on the second order superoperator master equation and consider in more detail the case when the time-dependent equation before projection arises in the interaction picture for some initial time-independent Liouvillian. In Section 2.2, we briefly discuss a superoperator analog of the Nakajima–Zwanzig equation as it is also used in open quantum systems [12].

In Section 3, we discuss a superoperator analog of a master equation that occurs in the stroboscopic limit of repeated non-selective measurements [13]. In particular, we discuss that actually there are several such analogs with different operational meanings. In Section 3.1, we focus on the second order master equation for the stroboscopic limit.

In Section 4, we introduce the averaging hyperprojector and summarize its properties. In Section 4.1, we discuss that its physical meaning is the transformation of dynamical maps as a result of the non-selective measurement of increments of the energy between two time moments. In particular, we show that the averaging hyperprojector is a map from quantum channels to quantum channels, i.e., a supermap or superchannel [14,15], which was discussed in the literature from different perspectives [16,17,18,19]. We also show that the averaging hyperprojector maps the unitary channel to the entropy non-decreasing channel. In Section 4.2, we show that the averaging hyperprojector can define the effective dynamics of fast observables, which were introduced in [8].

In Section 5, we consider the second order superoperator weak coupling master equation in the special case of the averaging hyperprojector. Using the expansion of the interaction Hamiltonian as a linear combination of eigenoperators of the commutator with the free Hamiltonian, we give more explicit representation of the second order generator. It is not necessarily of the Gorini–Kossakowski–Sudarshan–Lindblad (GKSL) form [20,21], but it is represented in the GKSL-like form. As usual, derivations of the Markovian master equations [22,23] and their corrections [24,25,26,27] are based not only on the weak coupling limit but on the Bogolubov–van Hove scaling [28,29]; as well, in Section 5.1 we discuss it for superoperator master equations. We also discuss its connection with algebraic perturbation theory [30,31,32,33,34].

In Section 6, we discuss the second order superoperator stroboscopic limit master equation for the averaging hyperprojector. In particular, we show that the entropy is non-decreasing in such a case.

In Conclusions, we summarize our results and discuss the directions of the further study.

## 2. Superoperator Weak Coupling Master Equation and Hyperprojector Technique

Let us briefly summarize a standard approach to the projection-based derivation of the quantum master equation. Let us consider a linear equation for a density matrix of the general form

(1) $$\frac{d}{dt}\rho \left(t\right)=\lambda L\left(t\right)\rho \left(t\right).$$

For the typical open quantum systems setup, for example, it is usually only the Liouville–von Neumann equation in the interaction picture for a system and a reservoir, but one can consider a more general setup of composite open quantum systems as well [35,36,37,38,39,40].

Furthermore, it is assumed that we are not interested in the full dynamics of ρ(t) but take an interest only in the projected dynamics Pρ(t) , where P is a projector (idempotent), i.e., $P^{2}=P$ .

There are several reasons why we are interested only in the projected dynamics. The first reason is the experimental accessibility. For example, in the open quantum systems setup, it is usually assumed that we have experimental access to open quantum systems only, but we have no experimental access to the heat bath degrees of freedom. We mostly follow this reason in our work. The second reason lies in simplifications of the projected dynamics compared to the full dynamics. For open quantum systems, typically the dynamics of the system only are simpler than the whole dynamics of the system and heat bath (at least after Markovian timescale separation). The third reason is that the given projector absorbs the physical assumptions in its structure. For the standard open quantum systems setup, it is an assumption of the approximately factorized state of the system and heat bath [41], (Paragraph 3.3.1). The fourth reason is that the projected dynamics are interesting for particular quantum technological applications [38,39]. Sometimes, even if we are experimentally interested in some degrees in addition to the projected ones, the projected dynamics can be an auxiliary computational tool [42].

Under weak coupling assumptions, we can write a master equation of the form [41], (Subsection 9.2.1)

(2) $$\frac{d}{dt}P\rho \left(t\right)=K\left(t\right)P\rho \left(t\right)+I\left(t\right)Q\rho \left(t_{0}\right),$$

where Q=I−P . We deal with time-convolutionless master equations, but in statistical physics, Nakajima–Zwanzig integro-differential equations could be usually derived. The inhomogeneous term $I\left(t\right)Q\rho \left(t_{0}\right)$ equals zero for the initial condition consistent with the projector, i.e., $P\rho \left(t_{0}\right)=\rho \left(t_{0}\right)$ . For open quantum system theory, the Argyres–Kelley [43] projector $P={\mathrm{Tr}}_{S}\left(\;\cdot \;\right)⊗\rho_{B}$ is typically used, and the consistency of the initial condition with the projector means that the initial state is factorized in this case. However, in general, Equation (2) has inhomogeneous terms [10]. Nevertheless, the adapted projection technique [11] can be used to obtain the homogeneous master equations. This technique assumes that the initial state can be approximated as a finite linear combination of the factorized matrices, and a separate homogeneous master equation is obtained for each factorized matrix.

Let us remark that the solution of (1) can always be written in the form

$$\rho \left(t\right)=\Phi_{t_{0}}^{t}\rho \left(t_{0}\right),$$

where $\Phi_{t_{0}}^{t}$ is a linear map, which is simply a Cauchy matrix in terms of linear ordinary differential equations theory, or a propagator, satisfying the propagator equation

$$\Phi_{t_{0}}^{t}=\Phi_{s}^{t}\Phi_{t_{0}}^{s}.$$

It is a unitary map for all *t* and $t_{0}$ if (1) is the Liouville–von Neumann equation and a completely positive map if (1) is the GKSL equation. The propagator $\Phi_{t_{0}}^{t}$ satisfies the equation

(3) $$\frac{d}{dt}\Phi_{t_{0}}^{t}=\lambda L\left(t\right)\Phi_{t_{0}}^{t},$$

which has the same form as Equation (1), but now it is an equation for superoperator $\Phi_{t_{0}}^{t}$ rather than operator ρ(t) , and it has the fixed initial condition

(4) $$\Phi_{t_{0}}^{t_{0}}=I,$$

while the initial condition $\rho (t_{0})$ for ρ(t) can be an arbitrary density matrix. Thus, it is natural to make the same procedure for $\Phi_{t_{0}}^{t}$ , deriving some analog of a master equation for the projected dynamics $P(\Phi_{t_{0}}^{t})$ . Here, P is now a projector that maps superoperators to superoperators, so we will call it the “hyperprojector”. Due to the initial condition (4), in contrast with master Equation (2), we can avoid inhomogeneity in the equation for $P(\Phi_{t_{0}}^{t})$ for all allowed $\Phi_{t_{0}}^{t}$ , if one assumes

(5) P(I)=I.

In such a case, we obtain the following superoperator analog of master Equation (2)

(6) $$\frac{d}{dt}P\left(\Phi_{t_{0}}^{t}\right)=K\left(t\right)P\left(\Phi_{t_{0}}^{t}\right).$$

In this section, we discuss the general setup, trying to emphasize the formal similarities and differences between operator and superoperator master equations. The general physical reasons to consider the projected dynamics are similar to the case of the usual projection superoperators mentioned above. In Section 4, we consider the specific hyperprojector and discuss concrete physical setups that lead to these hyperprojectors arising. Namely, in Section 4.2, we show that they naturally arise when one measures the correlation functions of the fast observables (fast oscillating time-dependent observables in resonance with a free Hamiltonian [8]), which are widely used in spectroscopy [44], (Section 4), [45], (Section 4), making only the projected dynamics experimentally accessible. As we will see in Section 4.1, it also arises as a non-selective measurement of energy (correspondent to free Hamiltonian).

The next proposition gives the perturbative expansion of K(t) .

**Proposition** **1.** *Asymptotic expansion at fixed t and for* K(t) *as* λ→0 *has the form*

(7) $$K\left(t\right)=\sum_{n=1}^{\infty }\lambda^{n}K_{n}\left(t\right),$$

*where* $K_{n}\left(t\right)$ *are defined as*

(8) $$K_{n}\left(t\right)\equiv \sum_{q=0}^{n-1}{\left(-1\right)}^{q}\sum_{\sum_{j=0}^{q}k_{j}=n,k_{j}⩾1}{\dot{M}}_{k_{0}}\left(t\right)M_{k_{1}}\left(t\right)\ldots M_{k_{q}}\left(t\right),$$

 *where the condition* $\sum_{j=0}^{q}k_{j}=n,k_{j}⩾1$ *means the sum runs over all compositions of the number n, and*

(9) $$M_{k}\left(t\right)\equiv \int_{t_{0}}^{t}dt_{k}\ldots \int_{t_{0}}^{t_{2}}dt_{1}P\left(L\left(t_{k}\right)\ldots L\left(t_{1}\right)\right),\;{\dot{M}}_{k}\left(t\right)\equiv \frac{d}{dt}M_{k}\left(t\right).$$

The proof follows the proof of Theorem 1 in [40] for a usual time-convolutionless master equation for the case when the initial condition is consistent with the projector.

For clarity, let us give explicit expressions of $K_{n}\left(t\right)$ for several first *n*.

(10) $$\begin{array}{cc} K_{1}\left(t\right)= & {\dot{M}}_{1}\left(t\right), \end{array}$$

(11) $$\begin{array}{cc} K_{2}\left(t\right)= & {\dot{M}}_{2}\left(t\right)-{\dot{M}}_{1}\left(t\right)M_{1}\left(t\right), \end{array}$$

(12) $$\begin{array}{cc} K_{3}\left(t\right)= & {\dot{M}}_{3}\left(t\right)-{\dot{M}}_{2}\left(t\right)M_{1}\left(t\right)-{\dot{M}}_{1}\left(t\right)M_{2}\left(t\right)+{\dot{M}}_{1}\left(t\right)M_{1}\left(t\right)M_{1}\left(t\right), \end{array}$$

(13) $$\begin{array}{cc} K_{4}\left(t\right)= & {\dot{M}}_{4}\left(t\right)-{\dot{M}}_{3}\left(t\right)M_{1}\left(t\right)-{\dot{M}}_{2}\left(t\right)M_{2}\left(t\right)-{\dot{M}}_{1}\left(t\right)M_{3}\left(t\right)\\ \; & +\;{\dot{M}}_{2}\left(t\right)M_{1}\left(t\right)M_{1}\left(t\right)+{\dot{M}}_{1}\left(t\right)M_{2}\left(t\right)M_{1}\left(t\right)+{\dot{M}}_{1}\left(t\right)M_{1}\left(t\right)M_{2}\left(t\right)-{\left(M_{1}\left(t\right)\right)}^{4}. \end{array}$$

Let us remark that we can also rewrite (8) using partially ordered cumulants similar to the Kubo–van Kampen partially ordered cumulants [46,47,48,49]. They can be derived similarly to the ones for the standard (Argyres–Kelley) projector with the only change of P·P in notation of [41], (Subsection 9.2.3) to P , i.e.,

(14) $$K_{n}\left(t\right)\equiv \int_{t_{0}}^{t}dt_{k}\ldots \int_{t_{0}}^{t_{2}}dt_{1}\kappa_{p.o.}^{P}\left(L\left(t\right)L\left(t_{k}\right)\ldots L\left(t_{1}\right)\right),$$

where

$$\begin{array}{cc} \; & \kappa_{p.o.}^{P}\left(L\left(t\right)L\left(t_{k}\right)\ldots L\left(t_{1}\right)\right)\\ \; & =\sum_{\sigma }\sum_{q=0}^{n-1}{\left(-1\right)}^{q}P\left(L\left(t\right)L\left(t_{\sigma (k)}\right)\ldots L\left(t_{\sigma (j_{q})}\right)\right)\\ \; & \;\times \;P\left(L\left(t_{\sigma (j_{q}-1)}\right)\ldots L\left(t_{\sigma (j_{q-1})}\right)\right)\ldots P\left(L\left(t_{\sigma (j_{1}-1)}\right)\ldots L\left(t_{\sigma (j_{1})}\right)\right). \end{array}$$

Here, the sum runs over all the permutations σ but with the restriction that inside any P , the terms are time-ordered, i.e., $t_{\sigma (k)}\geq t_{\sigma (k-1)}\geq \ldots \geq t_{\sigma (j_{q})}$ , $t_{\sigma (j_{q}-1)}\geq t_{\sigma (j_{q}-2)}\geq \ldots \geq t_{\sigma (j_{q-1})}$ , and so on.

Many other approaches that are used to calculate the perturbation theory terms of generators in usual master equations can be applied in our superoperator master setup as well. For example, the terms $K_{n}\left(t\right)$ can be calculated based on recurrence relations, similarly to [50]. Furthermore, the purely algebraic formulae for $M_{k}\left(t\right)$ can be obtained in the case when L(t) arises in the iteration picture for dynamics with a time-independent generator in the Schrödinger picture by taking all the integrals with respect to time, similarly to [40]. Their generalization to the superoperator master equation case is straightforward, so we will not dwell further on them.

### 2.1. Second Order Superoperator Master Equation

Second order master equations usually play the most important role in the theory of open quantum systems [41], (Sections 3.3–3.4), because the first order one is usually zero in the iteration picture or gives only the terms leading to unitary dynamics [51] and, therefore, has the same form as for closed systems. Therefore, for the superoperator master equation, we concentrate specifically on second order equations.

Taking into account (7), (10), (11) and (9), we obtain the following form of the generator in the second order of perturbation theory in λ .

(15) $$K\left(t\right)=\lambda P\left(L\left(t\right)\right)+\lambda^{2}\int_{t_{0}}^{t}ds\left(P\left(L\left(t\right)L\left(s\right)\right)-PL\left(t\right)PL\left(s\right)\right)+O\left(\lambda^{3}\right).$$

Let us consider the widespread case, where L(t) is Liouvillian in the interaction picture. Namely, we assume $L\left(t\right)=e^{-L_{0}\left(t-t_{0}\right)}Le^{L_{0}\left(t-t_{0}\right)}$ , where

(16) $$L_{0}=-i\left[H_{0},\;\cdot \;\right],\;L=-i\left[H_{I},\;\cdot \;\right],$$

with $H_{0}=H_{0}^{†}$ as a free Hamiltonian and $H_{I}=H_{I}^{†}$ as an interaction Hamiltonian. Then, taking the integral with respect to *s* in (15), similarly to [40], (Corollary 1), we have

(17) $$\begin{array}{cc} K(t)= & \lambda P(L(t))\\ \; & +\lambda^{2}\left(P\left(L\left(t\right){\left[L_{0},\;\cdot \;\right]}^{\left(-1\right)}\left(L\left(t_{0}\right)-L\left(t\right)\right)\right)-P\left(L\left(t\right)\right)P{\left[L_{0},\;\cdot \;\right]}^{\left(-1\right)}\left(L\left(t_{0}\right)-L\left(t\right)\right))\right)\\ \; & +O(\lambda^{3}). \end{array}$$

Here, ${\left[L_{0},\;\cdot \;\right]}^{\left(-1\right)}$ is a pseudoinverse such that

(18) $$\begin{array}{cc} \left[L_{0},\;\cdot \;\right]{\left[L_{0},\;\cdot \;\right]}^{\left(-1\right)} & ={\left[L_{0},\;\cdot \;\right]}^{\left(-1\right)}\left[L_{0},\;\cdot \;\right]=I-P_{\ker[L_{0},\;\cdot \;]}, \end{array}$$

(19) $$\begin{array}{cc} {}[L_{0}{,\;\cdot \;]}^{\left(-1\right)}P_{\ker[L_{0},\;\cdot \;]} & =P_{\ker[L_{0},\;\cdot \;]}{\left[L_{0},\;\cdot \;\right]}^{\left(-1\right)}=0, \end{array}$$

where $P_{\ker[L_{0},\;\cdot \;]}$ is a hyperprojector to the kernel of the map $\left[L_{0},\;\cdot \;\right]$ , i.e., it is zero on the kernel and the inverse in the usual sense on the orthogonal complement to the kernel. Such a pseudoinverse can be defined by the explicit formula [52], (Equation (2))

$${\left[L_{0},\;\cdot \;\right]}^{\left(-1\right)}\equiv \lim_{\varepsilon \to +0}\int_{0}^{\infty }ds\;e^{-\varepsilon s}\left(e^{-L_{0}s}\;\cdot \;e^{L_{0}s}\right)\left(I-P_{\ker[L_{0},\;\cdot \;]}\right).$$

Thus, within the accuracy of the second order of perturbation theory, the projected propagator ${\tilde{\Phi }}_{t_{0}}^{t}\equiv P\left(\Phi_{t_{0}}^{t}\right)$ can be defined as a solution of the Cauchy problem

(20) $$\begin{array}{cc} \; & \frac{d}{dt}{\tilde{\Phi }}_{t_{0}}^{t}=\lambda P\left(L\left(t\right)\right){\tilde{\Phi }}_{t_{0}}^{t}\\ \; & \;+\lambda^{2}\left(P\left(L\left(t\right){\left[L_{0},\;\cdot \;\right]}^{\left(-1\right)}\left(L\left(t_{0}\right)-L\left(t\right)\right)\right)-P\left(L\left(t\right)\right)P{\left[L_{0},\;\cdot \;\right]}^{\left(-1\right)}\left(L\left(t_{0}\right)-L\left(t\right)\right))\right){\tilde{\Phi }}_{t_{0}}^{t}, \end{array}$$

(21) $$\begin{array}{c} {\tilde{\Phi }}_{t_{0}}^{t_{0}}=I. \end{array}$$

### 2.2. Superoperator Nakajima-Zwanzig Equation

We are mostly interested in time-convolutionless superoperator master equations, but it is possible to obtain a superoperator analog of the Nakajima–Zwanzig equations as well. Due to condition (5), this analog also has the homogeneous form

(22) $$\frac{d}{dt}P\left(\Phi_{t_{0}}^{t}\right)=\lambda P\left(L\left(t\right)P\left(\Phi_{t_{0}}^{t}\right)\right)+\lambda^{2}\int_{t_{0}}^{t}ds\;P\left(L\left(t\right)G_{s}^{t}\left(\left(I-P\right)\left(L\left(s\right)P\left(\Phi_{t_{0}}^{s}\right)\right)\right)\right),$$

where $G_{s}^{t}$ is a map from superoperators to superoperators, which is defined as a solution of the Cauchy problem

(23) $$\frac{d}{dt}G_{s}^{t}=\lambda \left(I-P\right)\left(L\left(t\right)\;\cdot \;\right)G_{s}^{t},\;G_{s}^{s}=I.$$

Here, (L(t)·) is a map from superoperators to superoperators that acts as a left multiplication on superoperator L(t) .The derivation is similar to the one from the usual Nakajima–Zwanzig equation [41], (Subsection 9.1.2). By expanding (23) in the Dyson series, we can obtain perturbative expansion of the integral operator in the right-hand side of (22). Similarly to (14), we can represent the terms of this expansion as time-ordered integrals of (analogs of) Waldenfels cumulants [49]. Let us restrict ourselves to the second order Nakajima–Zwanzig superoperator equation. To obtain the second order terms in (22), we can simply use zero order terms in (23), i.e., only $G_{s}^{t}\equiv I+O\left(\lambda \right)$ . This leads to the following Cauchy problem for the second order Nakajima–Zwanzig equation for the projected propagator ${\tilde{\Phi }}_{t_{0}}^{t}\equiv P\left(\Phi_{t_{0}}^{t}\right)$

(24) $$\begin{array}{cc} \frac{d}{dt}{\tilde{\Phi }}_{t_{0}}^{t} & =\lambda P\left(L\left(t\right){\tilde{\Phi }}_{t_{0}}^{t}\right)+\lambda^{2}\int_{t_{0}}^{t}ds\;P\left(L\left(t\right)\left(I-P\right)\left(L\left(s\right){\tilde{\Phi }}_{t_{0}}^{s}\right)\right), \end{array}$$

(25) $$\begin{array}{cc} {\tilde{\Phi }}_{t_{0}}^{t_{0}} & =I. \end{array}$$

Sometimes [41], (Section 3.3.1), the usual second order time-convolutionless master equations are derived from the usual second order Nakajima–Zwanzig equation by assuming that the kernel of the analog of the integral operator in the right-hand side of (24) decays so fast when t−s differs from zero that one can take the density matrix at time *t* rather than *s* in this integral operator. However, if we assume a similar approximation here, i.e., that ${\tilde{\Phi }}_{t_{0}}^{s}\approx {\tilde{\Phi }}_{t_{0}}^{t}$ at the right-hand side of (24), we nevertheless do not obtain the superoperator master equation with generator (15). The difference is in the fact that ${\tilde{\Phi }}_{t_{0}}^{t}$ in (24) is inside of the action of the hyperprojector P , and this holds after the approximation ${\tilde{\Phi }}_{t_{0}}^{s}\approx {\tilde{\Phi }}_{t_{0}}^{t}$ , but in (20), it is outside of the action of the hyperprojector P . If we take, for example, P=P·P , where P is a usual projection superoperator, then this difference vanishes. However, let us emphasize that, in general, the second order superoperator Nakajima–Zwanzig Equation (24) with “Markovian approximation” ${\tilde{\Phi }}_{t_{0}}^{s}\approx {\tilde{\Phi }}_{t_{0}}^{t}$ is different from the second order time-convolutionless superoperator master equation (20). Therefore, in this aspect, our hyperprojector method and usual projection methods differ.

## 3. Stroboscopic Limit Superoperator Master Equation for Hyperprojectors

There are other master equations fully defined by projectors in addition to the weak coupling limit ones. Namely, the master equations that occur in the stroboscopic limit of repeated non-selective measurements [13]. Therefore, it is also natural to derive a superoperator analog from the master equations. In a usual setup, it is assumed that non-selective measurement is performed on an ancillary system (probe) at regular short time intervals. Furthermore, the system of interest and the ancillary system interact with each other and unitarily evolve between successive non-selective measurements.

In our setup, we do not need interaction with an ancillary system, but we assume that the measurement procedure can be modeled by a hyperprojector P (we see that it is the case in Section 4.1), and the evolution between measurements can be modeled by the quantum dynamical semigroup $\Phi_{t;\lambda }$

(26) $$\frac{d}{dt}\Phi_{t;\lambda }=\lambda L\Phi_{t;\lambda },\;\Phi_{0;\lambda }=I.$$

Following [13], we consider only the case of a time-independent generator. Then, evolution during small-time $N^{-1}t$ , N→∞ , but with strong coupling $\sqrt{N}\lambda$ (such a scaling is standard for the stroboscopic limit) is described by the map $\Phi_{N^{-1}t;\sqrt{N}\lambda }$ . If we perform a measurement P , starting before such an evolution and ending after it, then the resulting dynamical map has the form

$$P(\Phi_{N^{-1}t;\sqrt{N}\lambda }).$$

Now, if we perform such a procedure *N* times, then the total dynamical map has the form

(27) $$\underset{N\;\mathrm{times}}{\underset{︸}{P\left(\Phi_{N^{-1}t;\sqrt{N}\lambda }\right)\ldots P\left(\Phi_{N^{-1}t;\sqrt{N}\lambda }\right)}}={\left(P(\Phi_{N^{-1}t;\sqrt{N}\lambda })\right)}^{N}.$$

In the limit of an infinite number of such repetitions, it is a superoperator analog of the stroboscopic limit of repeated non-selective measurements [13], and it is described by the following proposition.

**Proposition** **2.** *Let* $\Phi_{t;\lambda }$ *be defined by (26). Then, for sufficiently large N*

(28) $${\left(P\left(\Phi_{N^{-1}t;\sqrt{N}\lambda }\right)\right)}^{N}=e^{L_{\mathrm{eff},\mathrm{str}}\left(N\right)t},$$

 *where* $L_{\mathrm{eff},\mathrm{str}}\left(N\right)$ *is the effective stroboscopic generator defined by asymptotic expansion*

(29) $$L_{\mathrm{eff},\mathrm{str}}\left(N\right)=\sum_{n=1}^{\infty }\lambda^{n}t^{n-1}N^{1-\frac{n}{2}}\sum_{k_{0}+\cdots +k_{m}=n}\frac{{\left(-1\right)}^{m}}{m+1}\frac{1}{k_{0}!\ldots k_{m}!}P\left(L^{k_{0}}\right)\cdots P\left(L^{k_{m}}\right)$$

 *as* N→∞ *.*

See Appendix A for the proof. Despite the fact that usually the stroboscopic limit assumes only second order expansion in λ , Proposition 2 allows one to calculate further perturbative correction up to arbitrary order.

Let us remark that there are other analogs of stroboscopic limit setups with a different order of measurements. As we discuss in Section 4.1, a particular hyperprojector P can model the non-selective measurement of increments of “usual” observables between two time moments. For such a hyperprojector, Formula (27) assumes that we measure the differences between each small time-interval of length $N^{-1}t$ . However, similarly, one can measure the increments between the initial time and successive time moments $nN^{-1}t$ , where n=1,…,N . Then, instead of (28), for sufficiently large *N*, we have

(30) $$\underset{N\;\mathrm{times}}{\underset{︸}{P(\Phi_{N^{-1}t;\sqrt{N}\lambda }P\left(\Phi_{N^{-1}t;\sqrt{N}\lambda }\cdots \right))}}=e^{L_{\mathrm{eff},\mathrm{str}}^{L}\left(N\right)t}.$$

Similarly, it is possible to consider a setup such that the final time moment for all two-time measurements is fixed, but initial moments are $nN^{-1}t$ , where n=0,…,N−1 . Then, instead of (28), for sufficiently large *N*, we have

(31) $$\underset{N\;\mathrm{times}}{\underset{︸}{P(\cdots P\left(P\left(\Phi_{N^{-1}t;\sqrt{N}\lambda }\right)\Phi_{N^{-1}t;\sqrt{N}\lambda }\right)\cdots )}}=e^{L_{\mathrm{eff},\mathrm{str}}^{R}\left(N\right)t}.$$

One can obtain perturbative expansions for $L_{\mathrm{eff},\mathrm{str}}^{R,L}\left(N\right)$ similar to the one given by (29), but they are more complicated so we do not provide general expressions for them, giving only the first and second terms of the expansions in the next subsection.

### 3.1. Second Order Superoperator Master Equation

Despite the fact that our perturbative expansion is in $N^{-\frac{1}{2}}$ , to emphasize the similarity to the weak coupling expansion of superoperator master equations in Section 2.1 we will call the order of perturbation of these equations in terms of order coupling constant λ . In particular, we call expansion (29), which holds the second order terms in λ the second order superoperator master equation. It has the form

(32) $$L_{\mathrm{eff},\mathrm{str}}\left(N\right)=\sqrt{N}\lambda P\left(L\right)+\frac{\lambda^{2}t}{2}\left(P\left(L^{2}\right)-{\left(P\left(L\right)\right)}^{2}\right)+O\left(N^{-\frac{1}{2}}\right)$$

as N→∞ .

Usually, the stroboscopic limit is formulated in terms of [13]

$$\gamma =\sqrt{N}\lambda ,\;\tau =\frac{t}{N},\;\gamma^{2}\tau =\lambda^{2}t=\mathrm{fix}.$$

In such a parametrization, the second order generator (32) is time-independent, and it takes the form

(33) $$L_{\mathrm{eff},\mathrm{str}}\left(N\right)=\gamma P\left(L\right)+\frac{\gamma^{2}\tau }{2}\left(P\left(L^{2}\right)-{\left(P\left(L\right)\right)}^{2}\right)+O\left(N^{-\frac{1}{2}}\right).$$

Thus, for $\Phi_{\mathrm{str}}=e^{tL_{\mathrm{eff},\mathrm{str}}\left(N\right)}$ we obtain, the following second order superoperator master equation

$$\frac{d}{dt}\Phi_{\mathrm{str},t}=\left(\gamma P\left(L\right)+\frac{\gamma^{2}\tau }{2}\left(P\left(L^{2}\right)-{\left(P\left(L\right)\right)}^{2}\right)\right)\Phi_{\mathrm{str},t},\;\Phi_{\mathrm{str},0}=I.$$

For the setups defined by Formulae (30)–(31), similarly to (32), we have

(34) $$L_{\mathrm{eff},\mathrm{str}}^{L}\left(N\right)=\sqrt{N}\lambda P\left(L\right)+\frac{\lambda^{2}t}{2}\left(P\left(LP\left(L\right)\right)-{\left(P\left(L\right)\right)}^{2}\right)+O\left(N^{-\frac{1}{2}}\right)$$

and

(35) $$L_{\mathrm{eff},\mathrm{str}}^{R}\left(N\right)=\sqrt{N}\lambda P\left(L\right)+\frac{\lambda^{2}t}{2}\left(P\left(P\left(L\right)L\right)-{\left(P\left(L\right)\right)}^{2}\right)+O\left(N^{-\frac{1}{2}}\right)$$

as N→∞ .

The difference between (32), (32) and (35) is similar to the difference between (15) and the time-convolutionless approximation of (24) discussed at the end of Section 2.2. Furthermore, it vanishes for P=P·P .

## 4. Averaging Hyperprojector

One of the first projections used to derive quantum master equations is the Zwanzig projection operator [53], which is nothing else but a particular case of the averaging projector [8] if the energy spectrum is non-degenerate. Therefore, it is natural to consider a hyperprojector analog of such a projector and the superoperator master equations for this specific hyperprojector.

The averaging (with respect to free dynamics $e^{L_{0}t}$ ) hyperprojector is defined by the formulae [9], (Definition 2.1)

(36) $$P\left(\Phi \right)=\lim_{T\to \infty }\frac{1}{T}\int_{0}^{T}dt\;e^{L_{0}t}\Phi e^{-L_{0}t},$$

where $L_{0}$ is the free Liouvillian defined by Formula (16), where $H_{0}$ is a free Hamiltonian, i.e., a Hermitian operator. Let us use the spectral decomposition of $H_{0}$ :

(37) $$H_{0}=\sum_{\varepsilon }\varepsilon \Pi_{\varepsilon },$$

where ε are (distinct) eigenvalues of $H_{0}$ , $\Pi_{\varepsilon }$ are orthogonal projections to the eigenspaces correspondent to these eigenvalues

(38) $$\Pi_{\varepsilon }\Pi_{\varepsilon^{\prime }}=\delta_{\varepsilon \varepsilon^{\prime }}\Pi_{\varepsilon },\;\Pi_{\varepsilon }=\Pi_{\varepsilon }^{†}.$$

The hyperprojector P can be represented as [9], (Proposition 2.1)

(39) $$P\left(\Phi \right)=\sum_{\varepsilon_{1}-\varepsilon_{2}=\varepsilon_{4}-\varepsilon_{3}}\Pi_{\varepsilon_{1}}\Phi \left(\Pi_{\varepsilon_{2}}\;\cdot \;\Pi_{\varepsilon_{3}}\right)\Pi_{\varepsilon_{4}}.$$

Let us summarize some other properties of P as the following proposition.

**Proposition** **3.** *The averaging hyperprojector* P *has the following properties:*

1. *Commuting of averaging with free dynamics*
(40) $$P\left(e^{L_{0}t}\Phi \right)=e^{L_{0}t}P\left(\Phi \right).$$
2. *Commuting of averaging **result** with free dynamics*
(41) $$e^{L_{0}t}P\left(\Phi \right)=P\left(\Phi \right)e^{L_{0}t}.$$
3. *Identity preserving property*
(42) P(I)=I,
  *where I is the identity superoperator.*
4. *Idempotent (projector) property*
(43) $$P^{2}=P.$$
5. *Unitality preservation*
(44) P(Φ)I=I
  *for any superoperator *Φ* such that* Φ(I)=I *, where I is the identity operator.*
6. *Coincidence with the hyperprojector to the kernel of the map* $\left[L_{0},\;\cdot \;\right]$
(45) $$P=P_{\ker[L_{0},\;\cdot \;]}.$$

See Appendix B for the proof. Properties 1 and 2 simplify the transformation from the interaction picture for projected dynamics. Property 3 is important as we assumed (4) to obtain homogeneous superoperator master equations. Property 4 means that P is indeed a hyperprojector. From property 5, it follows that, in particular, for unitary Φ (which is natural to start with), we have unital P(Φ) . The last property 6 gives an additional reason to consider such a hyperprojector due to the fact that it occurs anyway in the second order weak coupling superoperator master Equation (20) for an arbitrary hyperprojector, but through the definition (18)–(19) of the pseudoinverse ${\left[L_{0},\;\cdot \;\right]}^{\left(-1\right)}$ . Therefore, it is natural to consider the particular case when hyperprojectors P and $P_{\ker[L_{0},\;\cdot \;]}$ coincide.

### 4.1. Selective and Non-Selective Measurement of Increments

The averaging projector can be interpreted as a quantum operation correspondent to non-selective measurement (see, e.g., [54,55]) of energy (in the sense of the free Hamiltonian). In this subsection, we discuss that it is possible to interpret the averaging hyperprojector as non-selective measurement of increments of energy between two time moments without measuring the final and initial energies themselves.

Usually, multi-time measurements are discussed in terms of linear combinations of multi-time correlation functions (see discussion in [56], (Section 3.5.2)). Therefore, let us interpret the averaging hyperprojector in terms of them. For Markovian dynamics, in particular for the unitary dynamics of a closed system, a multi-time correlation function is defined by the generalized regression formula [41], (Section 3.2.4)

(46) $$\begin{array}{cc} \; & \langle Y^{\left(0\right)}\left(t_{0}\right)\cdot \cdots \cdot Y^{\left(N\right)}\left(t_{N}\right)X^{\left(N\right)}\left(t_{N}\right)\cdot \ldots \cdot X^{\left(0\right)}\left(t_{0}\right)\rangle \;\\ \; & \;=\mathrm{Tr}X^{\left(N\right)}\Phi_{t_{N-1}}^{t_{N}}\left(\ldots X^{\left(1\right)}\Phi_{t_{0}}^{t_{1}}\left(X^{\left(0\right)}\rho \left(t_{0}\right)Y^{\left(0\right)}\right)Y^{\left(1\right)}\ldots \right)Y^{\left(N\right)} \end{array}$$

for observables $Y^{\left(k\right)}$ and $X^{\left(k\right)}$ , k=0,⋯,N , and the time moments $t_{N}⩾\cdots ⩾t_{1}⩾t_{0}$ . Here $\Phi_{s}^{t}$ is defined by one-time dynamics $\rho \left(t\right)=\Phi_{s}^{t}\rho \left(s\right)$ , t⩾s . In general, the multi-time correlations can be non-Markovian and can be defined by process tensors [57,58], but here, we focus mostly on the Markovian case, for which all the multi-time correlations are fully defined by the propagator $\Phi_{s}^{t}$ and the initial density matrix. Thus, it is natural to consider the superoperator master equations for the projected propagator only. However, in principle, one can generalize the hyperprojector methods used here as multi-time hyperprojectors to derive multi-time master equations similar to Equations (27)–(28) in [59], where averaging with respect to a classical random variable played the role of such a multi-time hyperprojector.

For simplicity, let us consider the special case of Formula (46) for two-time measurements, which takes the form

(47) $$\langle Y\left(t\right)X\left(s\right)\rangle =\mathrm{Tr}Y\Phi_{s}^{t}\left(X\rho \left(s\right)\right),\;t⩾s.$$

Similarly to Formula (47), let us define

(48) $${\langle Y\left(t\right)X\left(s\right)\rangle }_{P}=\mathrm{Tr}YP\left(\Phi_{s}^{t}\right)\left(X\rho \left(s\right)\right),\;t⩾s.$$

Since we interpret hyperprojector P as a result of the non-selective measurement of increments of energy $H_{0}$ between times *s* and *t*, then it is natural to consider the hyperprojector defining the result of selective measurement as well. Therefore, we define

$$P_{\omega }\left(\Phi \right)=\sum_{\varepsilon_{1}-\varepsilon_{2}=\varepsilon_{4}-\varepsilon_{3}=\omega }\Pi_{\varepsilon_{1}}\Phi \left(\Pi_{\varepsilon_{2}}\;\cdot \;\Pi_{\varepsilon_{3}}\right)\Pi_{\varepsilon_{4}}$$

and the correspondent correlation function

$${\langle Y\left(t\right)X\left(s\right)\rangle }_{P_{\omega }}=\mathrm{Tr}YP_{\omega }\left(\Phi_{s}^{t}\right)\left(X\rho \left(s\right)\right),\;t⩾s.$$

The hyperprojectors $P_{\omega }$ sum up to P

(49) $$P=\sum_{\omega }P_{\omega }.$$

To verify that correlation functions ${\langle Y\left(t\right)X\left(s\right)\rangle }_{P}$ and ${\langle Y\left(t\right)X\left(s\right)\rangle }_{P_{\omega }}$ have operational meaning, let us show that they can be represented as linear combinations of the usual correlation functions (46).

**Proposition** **4.** *For arbitrary operators X and Y and* t⩾s *, one has*

(50) $$\begin{array}{cc} {\langle Y\left(t\right)X\left(s\right)\rangle }_{P_{\omega }} & =\sum_{\varepsilon_{1}-\varepsilon_{2}=\varepsilon_{4}-\varepsilon_{3}=\omega }\langle \Pi_{\varepsilon_{3}}\left(s\right)\Pi_{\varepsilon_{4}}\left(t\right)Y\left(t\right)\Pi_{\varepsilon_{1}}\left(t\right)\Pi_{\varepsilon_{2}}\left(s\right)X\left(s\right)\rangle , \end{array}$$

(51) $$\begin{array}{cc} {\langle Y\left(t\right)X\left(s\right)\rangle }_{P} & =\sum_{\varepsilon_{1}-\varepsilon_{2}=\varepsilon_{4}-\varepsilon_{3}}\langle \Pi_{\varepsilon_{3}}\left(s\right)\Pi_{\varepsilon_{4}}\left(t\right)Y\left(t\right)\Pi_{\varepsilon_{1}}\left(t\right)\Pi_{\varepsilon_{2}}\left(s\right)X\left(s\right)\rangle . \end{array}$$

See Appendix C for the proof. Moreover, analogously to Equations (50) and (51), we can define multi-time correlations, assuming that the generalized regression Formula (46) changes in a similar way, but only for the two times between which we perform the measurements. Such modified regression formulae fully define the process tensor [57,58,59].

Now, let us show that the hyperprojector $P_{\omega }$ can be interpreted as a hyperprojector of the (ideal) selective measurement of energy increment (only the increment without measurement of energies themselves). First of all, let us show that it can be used to define a probability of a certain value of the energy increment ω and the posterior state after such a measurement.

**Proposition** **5.** *If *Φ* is a completely positive trace non-increasing map, then* $P_{\omega }\left(\Phi \right)$ *is also a completely positive trace non-increasing map. (* $P_{\omega }$ *is a probabilistic supermap in terms of [14]). In particular, if ρ is a density matrix and *Φ* is a completely positive trace-preserving map, then*

1.
$\mathrm{Tr}(P_{\omega }\left(\Phi \right)\rho )$ *as a function of ω is a probability mass function, i.e.,*
(52) $$0⩽\mathrm{Tr}\left(P_{\omega }\left(\Phi \right)\rho \right)⩽1,\;\sum_{\omega }\mathrm{Tr}\left(P_{\omega }\left(\Phi \right)\rho \right)=1.$$
2. *If, in addition,* $\mathrm{Tr}(P_{\omega }\left(\Phi \right)\rho )\neq 0$ *, then*
(53) $$\frac{P_{\omega }\left(\Phi \right)\rho }{\mathrm{Tr}(P_{\omega }\left(\Phi \right)\rho )}$$
  *is a density matrix.*

See the proof in Appendix D. Thus, we can interpret $\mathrm{Tr}(P_{\omega }\left(\Phi \right)\rho )$ as a probability that some physical quantity equals ω , and the posterior state after such a measurement, if the result of the measurement is ω , is defined by (53).

Finally, the next proposition shows that $P_{\omega }$ satisfies several “natural” equalities that could be expected from the energy difference measurement hyperoperator.

**Proposition** **6.** *For arbitrary superoperator *Φ*, one has*

(54) $$\begin{array}{cc} P_{\varepsilon_{2}-\varepsilon_{1}}\left(\Phi \left(\Pi_{\varepsilon_{1}}\;\cdot \;\Pi_{\varepsilon_{1}}\right)\right) & =P_{\varepsilon_{2}-\varepsilon_{1}}\left(\Phi \right)\left(\Pi_{\varepsilon_{1}}\;\cdot \;\Pi_{\varepsilon_{1}}\right)=P_{\varepsilon_{2}-\varepsilon_{1}}\left(\left(\Pi_{\varepsilon_{2}}\;\cdot \;\Pi_{\varepsilon_{2}}\right)\Phi \right)\;\\ \; & =\left(\Pi_{\varepsilon_{2}}\;\cdot \;\Pi_{\varepsilon_{2}}\right)P_{\varepsilon_{2}-\varepsilon_{1}}\left(\Phi \right)=\left(\Pi_{\varepsilon_{2}}\;\cdot \;\Pi_{\varepsilon_{2}}\right)\Phi \left(\Pi_{\varepsilon_{1}}\;\cdot \;\Pi_{\varepsilon_{1}}\right). \end{array}$$

See the proof in Appendix E. Let us discuss the operational meaning of these equalities. It reflects the fact that measuring the energy and energy increment is equivalent to measuring both the initial and final energy. $P_{\varepsilon_{2}-\varepsilon_{1}}\left(\Phi \left(\Pi_{\varepsilon_{1}}\;\cdot \;\Pi_{\varepsilon_{1}}\right)\right)$ means that we started our experiment of measuring energy difference, then just after the start, we measured the energy; after that, the system evolved according to the propagator Φ , and we finished our experiment measuring the energy increment. $P_{\varepsilon_{2}-\varepsilon_{1}}\left(\Phi \right)\left(\Pi_{\varepsilon_{1}}\;\cdot \;\Pi_{\varepsilon_{1}}\right)$ means that we initially measured the energy and then ran the experiment on measuring the energy increment, and so on. $\left(\Pi_{\varepsilon_{2}}\;\cdot \;\Pi_{\varepsilon_{2}}\right)\Phi \left(\Pi_{\varepsilon_{1}}\;\cdot \;\Pi_{\varepsilon_{1}}\right)$ means that we just measured the energy at the initial and the final moment.

Now, let us consider non-selective measurement, i.e., we measure the energy increment but do not look at the result of the measurement. As we obtain state (53) with the probability $\mathrm{Tr}(P_{\omega }\left(\Phi \right)\rho )$ , then, after such a non-selective measurement, we have the following posterior state

$$\sum_{\omega }\mathrm{Tr}\left(P_{\omega }\left(\Phi \right)\rho \right)\frac{P_{\omega }\left(\Phi \right)\rho }{\mathrm{Tr}(P_{\omega }\left(\Phi \right)\rho )}=\sum_{\omega }P_{\omega }\left(\Phi \right)\rho =P\left(\Phi \right)\rho .$$

Thus, the hyperprojector P can be interpreted as a transformation of the dynamical map Φ between two time moments, which describes the action of non-selective energy increment measurement between these two time moments.

Proposition 5 also leads to some additional properties of hyperprojector P .

**Proposition** **7.**

1. *If *Φ* is a completely positive trace-preserving map (quantum channel), then* P(Φ) *is a completely positive trace-preserving map (quantum channel). (* P *is a deterministic supermap in terms of [14].)*
2. *If *Φ* is a unital (bistochastic) channel, i.e., *Φ* is a completely positive trace-preserving map and* Φ(I)=I *, then* P(Φ) *is a unital (bistochastic) channel as well.*

The usual averaging superoperator [8]

(55) $$P=\sum_{\varepsilon }\Pi_{\varepsilon }\;\cdot \;\Pi_{\varepsilon }$$

can be interpreted as the action of the usual non-selective measurement on a state, and in particular, it transforms density matrices before the measurement to density matrices after the measurement. Similarly, Proposition 7 says that Φ maps channels to channels and unital channels to unital channels. In particular, from [60], (Corollary 7.10), we obtain the following corollary.

**Corollary** **1.** *If* $\Phi =U\;\cdot \;U^{†}$ *, where U is a unitary matrix, then* P(Φ) *is entropy non-decreasing, i.e., for any density matrix ρ*

(56) S(P(Φ)ρ)⩾S(ρ),

 *where* S(ρ)=−Trρlogρ *is the von Neumann entropy.*

Let us remark that S(Φρ)=S(ρ) for $\Phi =U\;\cdot \;U^{†}$ . Thus, we have also a higher order analog of the H-theorem that can be interpreted as a higher order analog of the second law of thermodynamics from the physical point of view. The usual H-theorem says that entropy of a state increases (or does not change) after transformation by a bistochastic channel. However, here, we see that entropy increment increases (or does not change) if we transform the channel itself via the hyperprojector. It can be interpreted as a second order analog of the second law of thermodynamics if we change channels rather than states.

### 4.2. Correlation Functions of Fast Observables

In work [8], the usual averaging superoperator (55) was used to define the effective Gibbs state for fast observables. Let us show that, similarly, the averaging hyperprojector can be used to define the effective equilibrium correlation functions for these observables. By a fast observable, we mean an observable that explicitly depends on time in the Schrödinger picture in such a way that it becomes time-independent in the interaction picture. Namely, a fast observable X(t) has the following form in the Schrödinger picture [8], (Equation (3))

(57) $$X\left(t\right)=e^{-L_{0}t}\left(X\right),$$

where *X* is a constant matrix, where $L_{0}$ is defined by (16). Now, let us consider the equilibrium correlation function of two fast observables X(t) and Y(t) . By applying the generalized regression Formula (46), assuming that the initial state is a Gibbs state $\rho_{\beta }$ and taking into account the explicit time-dependence of fast observables, we have

$$\begin{array}{c} {\langle Y\left(t\right)X\left(s\right)\rangle }_{\mathrm{eq}}=\mathrm{Tr}e^{-L_{0}t}\left(Y\right)\Phi_{s}^{t}\left(e^{-L_{0}s}\left(X\right)\rho_{\beta }\right)=\mathrm{Tr}Ye^{L_{0}t}\Phi_{s}^{t}\left(e^{-L_{0}s}\left(X\right)\rho_{\beta }\right)\\ =\mathrm{Tr}Ye^{L_{0}t}\Phi_{s}^{t}e^{-L_{0}t}e^{L_{0}\left(t-s\right)}\left(Xe^{L_{0}s}\rho_{\beta }\right)). \end{array}$$

Similarly to [8], we consider a long timescale such that ωs≫1 for all non-zero Bohr frequencies ω and Δωt≫1 for all non-zero differences Δω in Bohr frequencies. However, we do not assume that t−s is large with respect to $\Delta \omega^{-1}$ or $\omega^{-1}$ . Then, on such a long timescale, we have

$$\begin{array}{cc} {\langle Y\left(t\right)X\left(s\right)\rangle }_{\mathrm{eq}} & \approx \mathrm{Tr}YP\left(\Phi_{s}^{t}\right)e^{L_{0}\left(t-s\right)}\left(XP\left(\rho_{\beta }\right)\right)\\ \; & =\mathrm{Tr}e^{-L_{0}\left(t-s\right)}\left(Y\right)P\left(\Phi_{s}^{t}\right)\left(XP\left(\rho_{\beta }\right)\right)\equiv {\langle Y\left(t-s\right)X\left(0\right)\rangle }_{\mathrm{eff}}. \end{array}$$

Thus, on such a long timescale, the correlation functions become approximately stationary, and instead of exact Gibbs state $\rho_{\beta }$ and dynamical map $\Phi_{s}^{t}$ , we can use the effective Gibbs state $P(\rho_{\beta })$ and $P(\Phi_{s}^{t})$ in regression Formula (47). Such an effective correlation function can be also considered as a multi-time analog of quasi-stationary states [61].

Similarly to [8], we can interpret the entropy increase in (56), in contrast to unitary dynamics, as the information being lost due to our restricted experimental capabilities, which assume that we can measure only “slow” averages of fast observables.

Let us also remark that in (57), *X* can be time-dependent but “slow” on the long timescale. We can think of *X* as a “slow envelope”.

## 5. Weak Coupling Superoperator Master Equation for Averaging Projector

Now, let us consider a second order weak coupling superoperator master equation in the special case of the averaging hyperprojector. Namely, let us consider unitary dynamics $\Psi_{t;\lambda }$ defined by equation

(58) $$\frac{d}{dt}\Psi_{t;\lambda }=\left(L_{0}+\lambda L_{I}\right)\Psi_{t;\lambda },\;\Psi_{t_{0};\lambda }=I.$$

The properties of the averaging hyperprojector simplify the transformation into and from the interaction picture.

**Proposition** **8.** *For projected propagator* $P(\Psi_{t;\lambda })$ *, the following Cauchy problem for the superoperator master equation holds*

(59) $$\frac{d}{dt}P\left(\Psi_{t;\lambda }\right)=L_{\mathrm{eff}}\left(t,t_{0};\lambda \right)P\left(\Psi_{t;\lambda }\right),\;P\left(\Psi_{t_{0};\lambda }\right)=I,$$

 *where*

(60) $$L_{\mathrm{eff}}\left(t,t_{0};\lambda \right)=L_{0}+\lambda P\left(L_{I}\right)+\lambda^{2}P\left(L_{I}{\left[L_{0},\;\cdot \;\right]}^{\left(-1\right)}\left(e^{\left(t-t_{0}\right)\left[L_{0},\;\cdot \;\right]}-I\right)L_{I}\right)+O\left(\lambda^{3}\right)$$

 *as* λ→0 *and fixed* $t⩾t_{0}$ *.*

See the proof in Appendix F.

Let us expand $H_{I}$ as a sum of eigenoperators of the superoperator $\left[H_{0},\;\cdot \;\right]$ in the same way as it is usually performed for the Markov master equation derivation [41], (Subsection 3.3.1), and use it to obtain more explicit representation of (60), as we have carried out for the effective (equilibrium) Hamiltonian in [8]. We have

(61) $$H_{I}=\sum_{\omega }D_{\omega },$$

where the sum runs over all the eigenvalues of $\left[H_{0},\;\cdot \;\right]$ , which are sometimes called the Bohr frequencies [23], p. 122 and

(62) $$\left[H_{0},D_{\omega }\right]=-\omega D_{\omega },\;D_{-\omega }=D_{\omega }^{†}.$$

The possibility to choose $D_{\omega }$ such that $D_{-\omega }=D_{\omega }^{†}$ follows from the fact that $H_{0}$ is Hermitian.

Then, Equation (60) takes the form (see Appendix G for detailed calculation)

(63) $$\begin{array}{cc} L_{\mathrm{eff}}\left(t,t_{0};\lambda \right)= & -i\left[H_{0}+\lambda D_{0}+\lambda^{2}\sum_{\omega \neq 0}\frac{1-\cos\omega (t-t_{0})}{\omega }D_{\omega }^{†}D_{\omega },\;\cdot \;\right]\;\\ \; & +2\lambda^{2}\sum_{\omega \neq 0}\frac{\sin\omega (t-t_{0})}{\omega }\left(D_{\omega }\;\cdot \;D_{\omega }^{†}-\frac{1}{2}\left\{D_{\omega }^{†}D_{\omega },\;\cdot \;\right\}\right)+O\left(\lambda^{3}\right). \end{array}$$

The first terms of this generator are nothing else but the commutator with the Hamiltonian in the rotation wave approximation $H_{0}+\lambda D_{0}=H_{\mathrm{RWA}}$ . The Hamiltonians obtained by perturbative corrections are usually called effective Hamiltonians [31,32]. In our case, we obtain not only Hamiltonian terms, so we call it an effective generator.

Let us remark that despite the fact that any Hermiticity and trace persevering generator has a GKSL-like form [62], the specific form (63) is such that Lindblad operators defined by (62) are the so-called weak coupling limit type (WCLT) form [63]. Therefore, our derivation shows that the averaging hyperprojector is the essential component for obtaining the weak coupling limit type form of generator. Actually, even in the derivation [22] of usual weak coupling master equations, we initially obtained a non-GKSL Redfield-like equation. However, the Redfield equation has a known physical issue in that it can predict non-positive probabilities in some cases [64]. Furthermore, only after using the averaging (but without regarding it as application of averaging hyperprojector explicitly), the WCLT GKSL generator occurs, which has no such issue. This suggests that hyperprojector methods can be essential for obtaining physically consistent equations.

Equation (63) has the GKSL-like form but not the GKSL form exactly, if

$$\frac{\sin\omega (t-t_{0})}{\omega }<0.$$

Hence, despite the fact that $P(\Psi_{t;\lambda })$ is completely positive and, due to corollary 1, is entropy non-decreasing from time $t_{0}$ to *t*, $P(\Psi_{t;\lambda })$ is not completely positive divisible and it is not necessarily entropy non-decreasing for any fixed time *t*. A more detailed description of the local entropy increase is given by the following proposition.

**Proposition** **9.**

1. *If* $\sin\omega (t-t_{0})⩾0$ *for all* $\omega \in \mathrm{spec}[H_{0},\;\cdot \;]$ *, such that* ω>0 *,*
(64) $$\frac{d}{dt}S\left(P\left(\Psi_{t;\lambda }\right)\rho \right)⩾0$$
*for all density matrices ρ and small enough λ.*
2. *If* $\sin\omega (t-t_{0})⩽0$ *for all* $\omega \in \mathrm{spec}[H_{0},\;\cdot \;]$ *, such that* ω>0 *,*
(65) $$\frac{d}{dt}S\left(P\left(\Psi_{t;\lambda }\right)\rho \right)⩽0$$
   *for all density matrices ρ and small enough λ.*

It follows from the fact that in the first case of this proposition generator, (63) has the GKSL form and $P(\Psi_{t;\lambda })$ is completely positive divisible and $L_{\mathrm{eff}}\left(t,t_{0};\lambda \right)I=0$ so it is unital, then by [60], (Corollary 7.10), we obtain (64). Similarly, in the second case of this proposition generator (63) has the opposite GKSL form and ${\left(P\left(\Psi_{t;\lambda }\right)\right)}^{-1}$ is completely positive divisible and unital, so we have (65).

This proposition describes some extreme situations, when the entropy increment at the time moment *t* has a definite sign for all the initial states. In general, it cannot be the case that $\sin\omega (t-t_{0})⩾0$ or $\sin\omega (t-t_{0})⩽0$ for all positive Bohr frequencies ω and the entropy increment at the time moment *t* can have different signs depending on state. However, the total entropy increment is non-negative by Corollary 1.

To illustrate (63), let us consider the simplest case of a two-level system

(66) $$H_{0}=\frac{1}{2}\omega_{0}\sigma_{z},\;H_{I}=g\sigma_{x},$$

then, (63) takes the form

(67) $$\begin{array}{cc} \; & L_{\mathrm{eff}}\left(t,t_{0};\lambda \right)=-i\left[\frac{1}{2}\omega_{0}\sigma_{z}+\lambda^{2}g^{2}\frac{1-\cos\omega_{0}\left(t-t_{0}\right)}{\omega_{0}}\sigma_{z},\;\cdot \;\right]\;\\ \; & +2\lambda^{2}g^{2}\frac{\sin\omega_{0}\left(t-t_{0}\right)}{\omega_{0}}\left(\sigma_{-}\;\cdot \;\sigma_{+}-\frac{1}{2}\left\{\sigma_{+}\sigma_{-},\;\cdot \;\right\}+\sigma_{+}\;\cdot \;\sigma_{-}-\frac{1}{2}\left\{\sigma_{-}\sigma_{+},\;\cdot \;\right\}\right)+O\left(\lambda^{3}\right). \end{array}$$

### 5.1. Bogolubov–Van Hove Limit

The mathematically strict derivation of Markovian master equations is based on the Bogolubov–van Hove scaling [22,23] and from the physical point of view the Bogolubov-van Hove limit identifies the timescale consistent with the projector. So let us discuss the Bogolubov–van Hove scaling analog in our case as well. We obtain the following proposition.

**Proposition** **10.** *Let* $L_{\mathrm{eff},\mathrm{BvH}}\left(\lambda \right)$ *be defined by*

$$\frac{d}{dt}P\left(\Psi_{\lambda^{-2}t;\lambda }\right)=L_{\mathrm{eff},\mathrm{BvH}}\left(\lambda \right)P\left(\Psi_{\lambda^{-2}t;\lambda }\right),\;\Psi_{t_{0};\lambda }=I,$$

 *then*

(68) $$L_{\mathrm{eff},\mathrm{BvH}}\left(\lambda \right)=\frac{1}{\lambda^{2}}L_{0}+\frac{1}{\lambda }P\left(L_{I}\right)-P\left(L_{I}{\left[L_{0},\;\cdot \;\right]}^{\left(-1\right)}L_{I}\right)+O\left(\lambda \right).$$

Using expansion (61)–(62), we obtain a more explicit form of the generator in the Bogolubov–van Hove limit:

(69) $$L_{\mathrm{eff},\mathrm{BvH}}\left(\lambda \right)=-i\left[\frac{1}{\lambda^{2}}H_{0}+\frac{1}{\lambda }D_{0}-\sum_{\omega >0}\frac{1}{\omega }\left[D_{\omega }^{†},D_{\omega }\right],\;\cdot \;\right]+O\left(\lambda \right).$$

Thus, after the Bogolubov–van Hove scaling in the limit λ→0 , the generator becomes a generator of fully unitary dynamics without any dissipator-like terms, which is not obvious from the abstract form (68). Let us also remark that there is a connection of Formula (69) with the algebraic perturbation theory [30,32,52]. The algebraic perturbation theory for given $L_{0}$ and $L_{I}$ finds perturbatively such U(λ) and $L_{\mathrm{eff},\sec}\left(\lambda \right)$ that

$$L_{0}+\lambda L_{I}=U^{-1}\left(\lambda \right)L_{\mathrm{eff},\sec}\left(\lambda \right)U\left(\lambda \right),$$

where $U\left(\lambda \right)=U\left(\lambda \right)\;\cdot \;U^{†}\left(\lambda \right)$ and U(λ) is a unitary transformation and $L_{\mathrm{eff},\sec}$ contains only the “secular” terms, which can be formalized [52] as the condition

$$L_{\mathrm{eff},\sec}=P\left(L_{\mathrm{eff},\sec}\right).$$

Comparing with Formula (13) from [52] for the effective Hamiltonian, we obtain

(70) $$L_{\mathrm{eff},\mathrm{BvH}}\left(\lambda \right)=\frac{1}{\lambda^{2}}L_{\mathrm{eff},\sec}\left(\lambda \right)+O\left(\lambda \right).$$

However, let us remark that after the Bogolubov–van Hove scaling, the unitary transformation U(λ) , which can also be found in [52], (Section IV.B)

$$U\left(\lambda \right)=\exp\left(\lambda \sum_{\omega >0}\left(D_{\omega }-D_{\omega }^{†}\right)\right)+O\left(\lambda^{2}\right),$$

seems to play no role. This is because $L_{\mathrm{eff},\mathrm{BvH}}\left(\lambda \right)$ in (69) has higher order corrections O(λ) , so with the same accuracy, U(λ)=I+O(λ) and the unitary transformation can be neglected. It is the result of the Bogolubov–van Hove scaling, but U(λ) plays the role for the higher order expansions of $L_{\mathrm{eff},\mathrm{BvH}}\left(\lambda \right)$ .

Therefore, despite the fact that the RWA (or secular approximation) and perturbative corrections to it are usually not formulated in terms of hyperprojectors and the Bogolubov–van Hove limit, these widespread physical assumptions can be compactly formulated via the hyperprojector method. For deriving systematic perturbative corrections, such a compact formulation can be important.

Let us also remark that from the point of view of interpretation discussed in Section 4.2, another analog of the Bogolubov–van Hove scaling can be considered, where both initial and final time are taken at the long timescale. This means that both *t* and $t_{0}$ are subjected to scaling. Although $\lambda^{-2}t$ and $\lambda^{-2}t_{0}$ are large as λ→0, it is possible to consider such sufficiently close times that $\lambda^{-2}\left(t-t_{0}\right)$ has a finite limit as λ→0 . Furthermore, no simplification occurs in (63) in such a case. However, the case when $\lambda^{-2}\left(t-t_{0}\right)$ is also large reduces to Proposition 10.

Let us also remark that in this work, we have focused on the finite-dimensional case. However, in the infinite-dimensional case, the sum in the dissipator-like term in (63) becomes the integral, which, similarly to [65], can give non-trivial terms in the limit due to the Fermi golden rule; therefore, it can be a non-trivial dissipator on the timescale identified by the Bogolubov–van Hove scaling. Owing to the connection with the algebraic perturbation theory mentioned in Section 5.1, it is also natural to ask if effective generators of unitary dynamics for the infinite-dimensional case always generate the unitary dynamics. This discussion allows us to hypothesize that possibly it is not the case. Some works interpreting resonances [66,67,68] in the infinite-dimensional case as spontaneous time-symmetry breaking [69] are also indirect evidence in favor of this hypothesis.

## 6. Stroboscopic Limit Superoperator Master Equation for Averaging Hyperprojector

Now, let us consider the stroboscopic limit superoperator master equation in the special case of the averaging hyperprojector. In an abstract form (32), it is not overly simplified in such a special case; therefore, we turn to a more explicit form. Using expansion (61) and (62), generator (32) takes the form

(71) $$\begin{array}{cc} \; & L_{\mathrm{eff},\mathrm{str}}\left(N\right)=-i\left[\sqrt{N}\lambda D_{0},\;\cdot \;\right]\;\\ \; & +\frac{\lambda^{2}t}{2}\sum_{\omega >0}\left(D_{\omega }\;\cdot \;D_{\omega }^{†}-\frac{1}{2}\left\{D_{\omega }^{†}D_{\omega },\;\cdot \;\right\}+D_{\omega }^{†}\;\cdot \;D_{\omega }-\frac{1}{2}\left\{D_{\omega }D_{\omega }^{†},\;\cdot \;\right\}\right)\;\\ \; & +O(N^{-\frac{1}{2}}). \end{array}$$

Let us remark that we hold the same notation $H_{0}$ , *H* and for expansion (61) and (62) to emphasize the similarity between the calculations. However, we do not have to understand $H_{0}$ as some Hamiltonian, but we could consider it as any other quantity. For the quantum thermodynamics applications, it can be volume or voltage or some other quantity, the increment of which we repeatedly measure.

Moreover, we can think about it not as a repeated measurement but as a constraint on dynamics, which indirectly describes the influence of the environment on an actually open system. For example, in non-quantum physics, we can just assume the ideal mechanical constraints or fixed voltage in a socket without describing a microscopic model of a surface or a rope that sets the ideal mechanical constraints and without a detailed model of a power plant that supplies the fixed voltage to the socket. In a certain sense, we also simply “project” unitary dynamics to constrained dynamics. Moreover, in our quantum case, the consistency with P for evolution between arbitrary close time moments can be considered as a constraints-based description of the environment’s influence on the system.

Generator (71) has the GKSL form. If we apply our projected dynamics to an initial density matrix, we can say that we have derived a quantum master equation without an explicit description of the reservoir, but only in terms of constraints on dynamics, which can be useful in the case where we do not have a detailed model of the reservoir and have access only to the system. To some extent, our approach is close to the discussion of the decoherence without a reservoir in [65].

As (71) is a generator of a unital semigroup [60], (Corollary 7.10), then we have the following proposition.

**Proposition** **11.** *For large enough N and arbitrary density matrix ρ, we have*

$$\frac{d}{dt}S\left(e^{L_{\mathrm{eff},\mathrm{str}}\left(N\right)t}\rho \right)⩾0.$$

To illustrate (71), let us also consider example (66), then (71) takes the form

$$L_{\mathrm{eff},\mathrm{str}}\left(N\right)=\frac{\lambda^{2}t}{2}g^{2}\left(\sigma_{-}\;\cdot \;\sigma_{+}-\frac{1}{2}\left\{\sigma_{+}\sigma_{-},\;\cdot \;\right\}+\sigma_{+}\;\cdot \;\sigma_{-}-\frac{1}{2}\left\{\sigma_{-}\sigma_{+},\;\cdot \;\right\}\right)+O\left(N^{-\frac{1}{2}}\right).$$

## 7. Conclusions

We have introduced superoperator master equations for the cases of the weak coupling limit and the stroboscopic limit in Section 2 and Section 3, respectively. We think that these equations can be interesting in a broad context, but in this work, we have focused on the particular case of the averaging hyperprojector. In Section 4.1, we have shown that it has physical meaning as a non-selective measurement of energy increments between two times without measuring the initial and final energies themselves. In Section 4.2, we have shown that it arises naturally if we measure the correlation functions of fast observables. It is important because in widely used spectroscopy setups [44], (Section 4), [45], (Section 4), we have no direct access to the density matrix but only to correlation functions. In particular, the multi-time correlations have now attracted great attention in open quantum systems theory [57,58,70]. Under the conditions of Corollary 1, we have also shown that such a hyperprojector leads to an increase in entropy increments, which can be regarded as the second order analog of the second law of thermodynamics. In Section 5 and Section 6, we apply the general approaches developed in Section 2 and Section 3 to the specific averaging hyperprojector. In Section 5, we have obtained the generator that has the WCLT GKSL-like form, which suggests that the averaging hyperprojector is essential for obtaining such a form. In Section 5.1, we have shown that in the Bogolubov–van Hove limit, this generator coincides with the second order effective generator from the algebraic perturbation theory, which suggests that RWA (or secular approximation) and its perturbative corrections can be absorbed by hyperprojector methods. In Section 6, we have derived the superoperator master equation arising due to the repeated measurement of increments of the observable that defines the averaging hyperprojector. It has the GKSL form and leads to dynamics with monotone entropy.

As a possible direction for further development, we should mention the consideration of analogs of other limits leading to Markovian master equations for open quantum systems, e.g., singular coupling limit [41], (Section 3.3.3), a singular and weak coupling limit combination leading to a unified master equation [71], low density limit [72,73,74] and so on. It can be interesting to find hyperprojectors that lead to the generators satisfying quantum detailed balance [75] rather than simply the unital, as in our work.

## Data Availability

Data are contained within the article.

## Abbreviations

The following abbreviations are used in this manuscript:

GKSL	Gorini–Kossakowski–Sudarshan–Lindblad
RWA	Rotating wave approximation
WCLT	Weak coupling limit type

## Appendix A. Stroboscopic Limit Expansion

The solution of (26) has the form $\Phi_{t;\lambda }=e^{\lambda Lt}$ , then the following asymptotic expansion holds

$$P\left(\Phi_{N^{-1}t;\sqrt{N}\lambda }\right)=P\left(e^{\frac{1}{\sqrt{N}}\lambda Lt}\right)=I+\sum_{k=1}^{\infty }\frac{\lambda^{k}t^{k}}{k!}N^{-\frac{k}{2}}P\left(L^{k}\right)$$

as N→∞ , where we have used (5). Then, using Formulae (A8) and (A12) from [8], we have

(A1)
$$\log P\left(\Phi_{N^{-1}t;\sqrt{N}\lambda }\right)=\sum_{n=0}^{\infty }\lambda^{n}t^{n}N^{-\frac{n}{2}}\sum_{k_{0}+\cdots +k_{m}=n}\frac{{\left(-1\right)}^{m}}{m+1}\frac{1}{k_{0}!\ldots k_{m}!}P\left(L^{k_{0}}\right)\cdots P\left(L^{k_{m}}\right)$$

Representing the left-hand side of Equation (28) in the form

$${\left(P\left(\Phi_{N^{-1}t;\sqrt{N}\lambda }\right)\right)}^{N}={\left(e^{\log P(\Phi_{N^{-1}t;\sqrt{N}\lambda })}\right)}^{N}=e^{t\frac{N}{t}\log P\left(\Phi_{N^{-1}t;\sqrt{N}\lambda }\right)},$$

we have $L_{\mathrm{eff},\mathrm{str}}\left(N\right)=\frac{N}{t}\log P\left(\Phi_{N^{-1}t;\sqrt{N}\lambda }\right)$ in the right-hand side of Equation (28). Taking into account (A1), we obtain (29).

## Appendix B. Proof for Properties of Averaging Projector

1. Taking into account (36), we have
  (A2)
  $$\begin{array}{c} P\left(e^{L_{0}t}\Phi \right)=\lim_{T\to \infty }\frac{1}{T}\int_{0}^{T}d\tau \;e^{L_{0}\tau }e^{L_{0}t}\Phi e^{-L_{0}\tau }=e^{L_{0}t}\lim_{T\to \infty }\frac{1}{T}\int_{0}^{T}d\tau \;e^{L_{0}\tau }\Phi e^{-L_{0}\tau }\\ =e^{L_{0}t}P\left(\Phi \right). \end{array}$$
  Thus, we obtain property 1.
2. Using (16), (39) and decomposition (37), we have
  (A3)
  $$\begin{array}{cc} e^{L_{0}t}P\left(\Phi \right) & =\sum_{\varepsilon_{1}-\varepsilon_{2}=\varepsilon_{4}-\varepsilon_{3}}e^{-iH_{0}t}\Pi_{\varepsilon_{1}}\Phi \left(\Pi_{\varepsilon_{2}}\;\cdot \;\Pi_{\varepsilon_{3}}\right)\Pi_{\varepsilon_{4}}e^{iH_{0}t}\;\\ \; & =\sum_{\varepsilon_{1}-\varepsilon_{4}=\varepsilon_{2}-\varepsilon_{3}}e^{-i\varepsilon_{1}t}\Pi_{\varepsilon_{1}}\Phi \left(\Pi_{\varepsilon_{2}}\;\cdot \;\Pi_{\varepsilon_{3}}\right)\Pi_{\varepsilon_{4}}e^{i\varepsilon_{4}t}\;\\ \; & =\sum_{\varepsilon_{1}-\varepsilon_{4}=\varepsilon_{2}-\varepsilon_{3}}\Pi_{\varepsilon_{1}}\Phi \left(\Pi_{\varepsilon_{2}}e^{-i\varepsilon_{2}t}\;\cdot \;e^{i\varepsilon_{3}t}\Pi_{\varepsilon_{3}}\right)\Pi_{\varepsilon_{4}}\;\\ \; & =\sum_{\varepsilon_{1}-\varepsilon_{4}=\varepsilon_{2}-\varepsilon_{3}}\Pi_{\varepsilon_{1}}\Phi \left(\Pi_{\varepsilon_{2}}e^{-iH_{0}t}\;\cdot \;e^{iH_{0}t}\Pi_{\varepsilon_{3}}\right)\Pi_{\varepsilon_{4}}=P\left(\Phi \right)e^{L_{0}t}. \end{array}$$
  Thus, we obtain property 2.
3. Taking into account (36), we have
  (A4)
  $$P\left(I\right)=\lim_{T\to \infty }\frac{1}{T}\int_{0}^{T}d\tau \;e^{L_{0}\tau }Ie^{-L_{0}\tau }=\lim_{T\to \infty }\frac{1}{T}\int_{0}^{T}d\tau I=I.$$
  Thus, we obtain property 3.
4. Using property 2, we have
  (A5)
  $$e^{L_{0}t}P\left(\Phi \right)e^{-L_{0}t}=P\left(\Phi \right),$$
  then
  (A6)
  $$P^{2}\left(\Phi \right)=\lim_{T\to \infty }\frac{1}{T}\int_{0}^{T}d\tau \;e^{L_{0}\tau }P\left(\Phi \right)e^{-L_{0}\tau }=\lim_{T\to \infty }\frac{1}{T}\int_{0}^{T}d\tau \;P\left(\Phi \right)=P\left(\Phi \right).$$
  Thus, we obtain property 4.
5. Using (39) and (38), we have
  $$P\left(\Phi \right)\left(I\right)=\sum_{\varepsilon_{1}-\varepsilon_{2}=\varepsilon_{4}-\varepsilon_{3}}\Pi_{\varepsilon_{1}}\Phi \left(\Pi_{\varepsilon_{2}}\Pi_{\varepsilon_{3}}\right)\Pi_{\varepsilon_{4}}=\sum_{\varepsilon_{1},\varepsilon_{2}}\Pi_{\varepsilon_{1}}\Phi \left(\Pi_{\varepsilon_{2}}\right)\Pi_{\varepsilon_{1}}=\sum_{\varepsilon_{1}}\Pi_{\varepsilon_{1}}\Phi \left(I\right)\Pi_{\varepsilon_{1}}.$$
  If Φ(I)=I , then we have
  $$P\left(\Phi \right)\left(I\right)=\sum_{\varepsilon_{1}}\Pi_{\varepsilon_{1}}^{2}=\sum_{\varepsilon_{1}}\Pi_{\varepsilon_{1}}=I.$$
  Thus, we obtain property 5.
6. Let us assume $\Phi \in \ker[L_{0},\;\cdot \;]$ , i.e., $[L_{0},\;\cdot \;]\Phi =0$ . Then, $e^{[L_{0},\;\cdot \;]t}\Phi =\Phi$ and using definition (36), we have
  $$P\left(\Phi \right)=\lim_{T\to \infty }\frac{1}{T}\int_{0}^{T}dt\;e^{[L_{0},\;\cdot \;]t}\Phi =\lim_{T\to \infty }\frac{1}{T}\int_{0}^{T}dt\;\Phi =\Phi ,$$
  Now, conversely, let us assume P(Φ)=Φ , then due to (A5) we have
  $$e^{[L_{0},\;\cdot \;]t}\Phi =e^{[L_{0},\;\cdot \;]t}P\left(\Phi \right)=P\left(\Phi \right)=\Phi .$$
  Differentiating both sides at t=0 , we have
  $$\left[L_{0},\;\cdot \;\right]\Phi ={\left.\frac{d}{dt}e^{[L_{0},\;\cdot \;]t}\Phi \right|}_{t=0}=0.$$
  Therefore, we have $\Phi \in \ker[L_{0},\;\cdot \;]$ . Thus, we obtain property 6.

## Appendix C. Correlation Functions for Projected Propagators

The generalized regression Formula (46) gives

$$\langle \Pi_{\varepsilon_{3}}\left(s\right)\Pi_{\varepsilon_{4}}\left(t\right)Y\left(t\right)\Pi_{\varepsilon_{1}}\left(t\right)\Pi_{\varepsilon_{2}}\left(s\right)X\left(s\right)\rangle =\mathrm{Tr}\left(Y\Pi_{\varepsilon_{1}}\Phi_{s}^{t}\left(\Pi_{\varepsilon_{2}}X\rho \left(s\right)\Pi_{\varepsilon_{3}}\right)\Pi_{\varepsilon_{4}}\right).$$

Therefore, we have

$$\begin{array}{cc} \; & \sum_{\varepsilon_{1}-\varepsilon_{2}=\varepsilon_{4}-\varepsilon_{3}=\omega }\langle \Pi_{\varepsilon_{3}}\left(s\right)\Pi_{\varepsilon_{4}}\left(t\right)Y\left(t\right)\Pi_{\varepsilon_{1}}\left(t\right)\Pi_{\varepsilon_{2}}\left(s\right)X\left(s\right)\rangle \\ \; & \;=\sum_{\varepsilon_{1}-\varepsilon_{2}=\varepsilon_{4}-\varepsilon_{3}=\omega }\mathrm{Tr}\left(Y\Pi_{\varepsilon_{1}}\Phi_{s}^{t}\left(\Pi_{\varepsilon_{2}}X\rho \left(s\right)\Pi_{\varepsilon_{3}}\right)\Pi_{\varepsilon_{4}}\right)=\mathrm{Tr}\left(YP_{\omega }\left(\Phi_{s}^{t}\right)\left(X\rho \left(s\right)\right)\right) \end{array}$$

and we obtain (50). Then, using (49), we have

$$\begin{array}{cc} \mathrm{Tr}(YP\left(\Phi_{s}^{t}\right)\left(X\rho \left(s\right)\right)) & =\sum_{\omega }\mathrm{Tr}\left(YP_{\omega }\left(\Phi_{s}^{t}\right)\left(X\rho \left(s\right)\right)\right)\\ \; & =\sum_{\omega }\sum_{\varepsilon_{1}-\varepsilon_{2}=\varepsilon_{4}-\varepsilon_{3}=\omega }\langle \Pi_{\varepsilon_{3}}\left(s\right)\Pi_{\varepsilon_{4}}\left(t\right)Y\left(t\right)\Pi_{\varepsilon_{1}}\left(t\right)\Pi_{\varepsilon_{2}}\left(s\right)X\left(s\right)\rangle \\ \; & =\sum_{\varepsilon_{1}-\varepsilon_{2}=\varepsilon_{4}-\varepsilon_{3}}\langle \Pi_{\varepsilon_{3}}\left(s\right)\Pi_{\varepsilon_{4}}\left(t\right)Y\left(t\right)\Pi_{\varepsilon_{1}}\left(t\right)\Pi_{\varepsilon_{2}}\left(s\right)X\left(s\right)\rangle . \end{array}$$

Thus, we obtain (51).

## Appendix D. Properties of Increment Hyperprojector

If Φ is completely positive, then it has Kraus representation [60], (Corollary 6.13)

$$\Phi =\sum_{j}W_{j}\;\cdot \;W_{j}^{†},$$

where $W_{j}$ are Kraus operators. Using this allows us to calculate

$$\begin{array}{cc} P_{\varepsilon }\left(\Phi \right)\rho & =\sum_{\varepsilon_{1}-\varepsilon_{2}=\varepsilon_{4}-\varepsilon_{3}=\varepsilon }\sum_{j}\Pi_{\varepsilon_{1}}W_{j}\Pi_{\varepsilon_{2}}\rho \Pi_{\varepsilon_{3}}W_{j}^{†}\Pi_{\varepsilon_{4}}\\ \; & =\sum_{j}\sum_{\varepsilon_{1}-\varepsilon_{2}=\varepsilon }\Pi_{\varepsilon_{1}}W_{j}\Pi_{\varepsilon_{2}}\rho \sum_{\varepsilon_{4}-\varepsilon_{3}=\varepsilon }\Pi_{\varepsilon_{3}}W_{j}^{†}\Pi_{\varepsilon_{4}}. \end{array}$$

As

$${\left(\sum_{\varepsilon_{1}-\varepsilon_{2}=\varepsilon }\Pi_{\varepsilon_{1}}W_{j}\Pi_{\varepsilon_{2}}\right)}^{†}=\sum_{\varepsilon_{1}-\varepsilon_{2}=\varepsilon }\Pi_{\varepsilon_{2}}W_{j}^{†}\Pi_{\varepsilon_{1}}=\sum_{\varepsilon_{4}-\varepsilon_{3}=\varepsilon }\Pi_{\varepsilon_{3}}W_{j}^{†}\Pi_{\varepsilon_{4}},$$

then we obtain Kraus representation for $P_{\varepsilon }$

$$P_{\varepsilon }\left(\Phi \right)=\sum_{j}W_{\varepsilon ,j}\;\cdot \;W_{\varepsilon ,j}^{†},$$

where

(A7)
$$W_{\varepsilon ,j}=\sum_{\varepsilon_{1}-\varepsilon_{2}=\varepsilon }\Pi_{\varepsilon_{1}}W_{j}\Pi_{\varepsilon_{2}}.$$

Therefore, $P_{\varepsilon }\left(\Phi \right)$ is a completely positive map. In particular, $P_{\varepsilon }\left(\Phi \right)\rho ⩾0$ if ρ is a density matrix. Therefore, if $\mathrm{Tr}P_{\varepsilon }\left(\Phi \right)\rho \neq 0$ , then (53) is a density matrix.

If Φ is a completely positive trace non-increasing map, then Kraus operators $W_{j}$ satisfy [76], (Theorem 8.1)

$$\sum_{j}W_{j}^{†}W_{j}⩽I.$$

Then, taking into account (A7), we have

$$\begin{array}{cc} \sum_{j}W_{\varepsilon ,j}^{†}W_{\varepsilon ,j} & =\sum_{j}\sum_{\varepsilon_{4}-\varepsilon_{3}=\varepsilon }\Pi_{\varepsilon_{3}}W_{j}^{†}\Pi_{\varepsilon_{4}}\sum_{\varepsilon_{1}-\varepsilon_{2}=\varepsilon }\Pi_{\varepsilon_{1}}W_{j}\Pi_{\varepsilon_{2}}\\ \; & =\sum_{j}\sum_{\varepsilon_{4}-\varepsilon_{3}=\varepsilon }\sum_{\varepsilon_{1}-\varepsilon_{2}=\varepsilon }\Pi_{\varepsilon_{3}}W_{j}^{†}\Pi_{\varepsilon_{4}}\Pi_{\varepsilon_{1}}W_{j}\Pi_{\varepsilon_{2}}\\ \; & =\sum_{j}\sum_{\varepsilon_{1}-\varepsilon_{2}=\varepsilon }\Pi_{\varepsilon_{2}}W_{j}^{†}\Pi_{\varepsilon_{1}}W_{j}\Pi_{\varepsilon_{2}}⩽\sum_{j}\sum_{\varepsilon_{1},\varepsilon_{2}}\Pi_{\varepsilon_{2}}W_{j}^{†}\Pi_{\varepsilon_{1}}W_{j}\Pi_{\varepsilon_{2}}\\ \; & =\sum_{j}\sum_{\varepsilon_{2}}\Pi_{\varepsilon_{2}}W_{j}^{†}W_{j}\Pi_{\varepsilon_{2}}⩽\sum_{\varepsilon_{2}}\Pi_{\varepsilon_{2}}\Pi_{\varepsilon_{2}}=\sum_{\varepsilon_{2}}\Pi_{\varepsilon_{2}}=I \end{array}$$

Thus, $P_{\varepsilon }\left(\Phi \right)$ is trace non-increasing if Φ is. In particular, $\mathrm{Tr}(P_{\varepsilon }\left(\Phi \right)\rho )⩽1$ . Therefore, we obtain (52).

## Appendix E. Increment Hyperprojector and Energy Measurement

Using (38) and (39), we have

$$\begin{array}{cc} P_{\varepsilon_{2}-\varepsilon_{1}}\left(\Phi \left(\Pi_{\varepsilon_{1}}\;\cdot \;\Pi_{\varepsilon_{1}}\right)\right) & =\sum_{\varepsilon_{1}^{\prime }-\varepsilon_{2}^{\prime }=\varepsilon_{4}^{\prime }-\varepsilon_{3}^{\prime }=\varepsilon_{2}-\varepsilon_{1}}\Pi_{\varepsilon_{1}^{\prime }}\Phi \left(\Pi_{\varepsilon_{1}}\Pi_{\varepsilon_{2}^{\prime }}\;\cdot \;\Pi_{\varepsilon_{3}^{\prime }}\Pi_{\varepsilon_{1}}\right)\Pi_{\varepsilon_{4}^{\prime }}\\ \; & =\sum_{\varepsilon_{1}^{\prime }-\varepsilon_{2}^{\prime }=\varepsilon_{4}^{\prime }-\varepsilon_{3}^{\prime }=\varepsilon_{2}-\varepsilon_{1}}\Pi_{\varepsilon_{1}^{\prime }}\Phi \left(\delta_{\varepsilon_{1},\varepsilon_{2}^{\prime }}\Pi_{\varepsilon_{1}}\;\cdot \;\delta_{\varepsilon_{1},\varepsilon_{3}^{\prime }}\Pi_{\varepsilon_{1}}\right)\Pi_{\varepsilon_{4}^{\prime }}\\ \; & =\sum_{\varepsilon_{1}^{\prime }-\varepsilon_{1}=\varepsilon_{4}^{\prime }-\varepsilon_{1}=\varepsilon_{2}-\varepsilon_{1}}\Pi_{\varepsilon_{1}^{\prime }}\Phi \left(\Pi_{\varepsilon_{1}}\;\cdot \;\Pi_{\varepsilon_{1}}\right)\Pi_{\varepsilon_{4}^{\prime }}\\ \; & =\sum_{\varepsilon_{1}^{\prime }=\varepsilon_{4}^{\prime }=\varepsilon_{2}}\Pi_{\varepsilon_{1}^{\prime }}\Phi \left(\Pi_{\varepsilon_{1}}\;\cdot \;\Pi_{\varepsilon_{1}}\right)\Pi_{\varepsilon_{4}^{\prime }}=\Pi_{\varepsilon_{2}}\Phi \left(\Pi_{\varepsilon_{1}}\;\cdot \;\Pi_{\varepsilon_{1}}\right)\Pi_{\varepsilon_{2}}. \end{array}$$

Thus, we obtain the equality between the first and last expressions in (54). The equality between the other expressions in (54) and the last one can be proven in a similar way.

## Appendix F. Second Order Weak Coupling Generator

Let us move to the interaction picture. Namely, if we define $\Phi_{t_{0}}^{t}=e^{-L_{0}\left(t-t_{0}\right)}\Psi_{t;\lambda }$ , then we obtain the equation of form (3) and initial condition (4), where $L\left(t\right)=e^{-L_{0}\left(t-t_{0}\right)}L_{I}e^{L_{0}\left(t-t_{0}\right)}$ . Taking into account properties 1 and 2 from Proposition 3, we have

(A8)
$$P\left(L\left(t\right)\right)=P\left(e^{-L_{0}\left(t-t_{0}\right)}L_{I}e^{L_{0}\left(t-t_{0}\right)}\right)=P\left(L_{I}\right)$$

and

(A9)
$$\begin{array}{cc} P(L(t)L(s)) & =P(e^{-L_{0}\left(t-t_{0}\right)}L_{I}e^{L_{0}\left(t-t_{0}\right)}e^{-L_{0}\left(s-t_{0}\right)}L_{I}e^{L_{0}\left(s-t_{0}\right)})\;\\ \; & =P\left(L_{I}e^{L_{0}\left(t-s\right)}L_{I}e^{-L_{0}\left(t-s\right)}\right)=P\left(L_{I}e^{\left[L_{0},\;\cdot \;\right]\left(t-s\right)}\left(L_{I}\right)\right). \end{array}$$

Therefore,

(A10)
$$\begin{array}{cc} P\left(L\left(t\right){\left[L_{0},\;\cdot \;\right]}^{\left(-1\right)}\left(L\left(t_{0}\right)-L\left(t\right)\right)\right)\; & =\int_{t_{0}}^{t}ds(P\left(L\left(t\right)L\left(s\right)\right)\;\\ \; & =P\left(L_{I}\frac{e^{\left(t-t_{0}\right)\left[L_{0},\;\cdot \;\right]}-1}{\left[L_{0},\;\cdot \;\right]}\left(L_{I}\right)\right)\;\\ \; & =P\left(L_{I}{\left[L_{0},\;\cdot \;\right]}^{\left(-1\right)}\left(e^{\left(t-t_{0}\right)\left[L_{0},\;\cdot \;\right]}-I\right)L_{I}\right). \end{array}$$

Due to property 6 from Proposition 3 and definition (19) of the pseudoinverse, we have

(A11)
$$P{\left[L_{0},\;\cdot \;\right]}^{\left(-1\right)}\left(L\left(t_{0}\right)-L\left(t\right)\right)=0.$$

Substituting (A8), (A10) and (A11) to (17) and using property 1 from Proposition 3 to return from the interaction picture of projected dynamics $P(\Phi_{t_{0}}^{t})$ to the Schrödinger one $P(\Psi_{t;\lambda })$ , we obtain (60).

## Appendix G. Explicit Form of Second Order Weak Coupling Generator

From (16) and (62), we have

$$L_{0}\left(D_{\omega }\right)=i\omega D_{\omega }.$$

Then, by the Leibniz rule for a commutator, we obtain

$$L_{0}\left(D_{\omega }X\right)=L_{0}\left(D_{\omega }\right)X+D_{\omega }L_{0}\left(X\right)=i\omega D_{\omega }X+D_{\omega }L_{0}\left(X\right)$$

for arbitrary operator *X*. It can be written as

$$\left[L_{0},D_{\omega }\;\cdot \;\right]=i\omega D_{\omega }\;\cdot \;,$$

or, equivalently, as

(A12)
$$\left[L_{0},\;\cdot \;\right]\left(D_{\omega }\;\cdot \;\right)=i\omega \left(D_{\omega }\;\cdot \;\right).$$

Similarly, we have

(A13)
$$\left[L_{0},\;\cdot \;\right]\left(\;\cdot \;D_{\omega }\right)=i\omega \left(\;\cdot \;D_{\omega }\right).$$

By combining Formulae (A12) and (A13), we obtain

$$\left[L_{0},\;\cdot \;\right]\left[D_{\omega },\;\cdot \;\right]=i\omega \left[D_{\omega },\;\cdot \;\right],$$

from which we have

$$e^{[L_{0},\;\cdot \;]t}\left[D_{\omega },\;\cdot \;\right]=e^{i\omega t}\left[D_{\omega },\;\cdot \;\right].$$

Using expansion (61), we have

(A14)
$$e^{[L_{0},\;\cdot \;]t}L_{I}=-ie^{[L_{0},\;\cdot \;]t}\left[H_{I},\;\cdot \;\right]=-i\sum_{\omega }e^{i\omega t}\left[D_{\omega },\;\cdot \;\right].$$

Then

$$\begin{array}{cc} \; & P\left(L_{I}{\left[L_{0},\;\cdot \;\right]}^{\left(-1\right)}\left(e^{\left(t-t_{0}\right)\left[L_{0},\;\cdot \;\right]}-I\right)L_{I}\right)\\ \; & =-iP\left(L_{I}\sum_{\omega \neq 0}\frac{e^{i\omega (t-t_{0})}-1}{\omega }\left[D_{\omega },\;\cdot \;\right]\right)\\ \; & =-P\left(\sum_{\omega^{\prime }}\sum_{\omega \neq 0}\frac{e^{i\omega (t-t_{0})}-1}{\omega }\left[D_{\omega^{\prime }},\left[D_{\omega },\;\cdot \;\right]\right]\right)\\ \; & =-\sum_{\omega \neq 0}\frac{e^{i\omega (t-t_{0})}-1}{\omega }\left[D_{\omega }^{†},\left[D_{\omega },\;\cdot \;\right]\right]\\ \; & =-i\left[\sum_{\omega \neq 0}\frac{1-\cos\omega (t-t_{0})}{\omega }D_{\omega }^{†}D_{\omega },\;\cdot \;\right]\\ \; & +2\sum_{\omega \neq 0}\frac{\sin\omega (t-t_{0})}{\omega }\left(D_{\omega }\;\cdot \;D_{\omega }^{†}-\frac{1}{2}\left\{D_{\omega }^{†}D_{\omega },\;\cdot \;\right\}\right) \end{array}$$

and

$$P\left(L_{I}\right)=P\left(-i\sum_{\omega }\left[D_{\omega },\;\cdot \;\right]\right)=-i\sum_{\omega }\left[D_{0},\;\cdot \;\right].$$

Thus, (60) takes form (63).
